# Pre- and Post-Resection Urine Metabolic Profiles of Bladder Cancer Patients: Results of Preliminary Studies on Time Series Metabolomics Analysis

**DOI:** 10.3390/cancers14051210

**Published:** 2022-02-25

**Authors:** Julia Jacyna, Marta Kordalewska, Małgorzata Artymowicz, Marcin Markuszewski, Marcin Matuszewski, Michał J. Markuszewski

**Affiliations:** 1 Department of Biopharmaceutics and Pharmacodynamics, Medical University of Gdańsk, Al. Gen. J. Hallera 107, 80-416 Gdańsk, Poland; marta.kordalewska@gumed.edu.pl (M.K.); malgorzata.artymowicz@gumed.edu.pl (M.A.); 2 University Clinical Center, Hospital Pharmacy, M. Smoluchowskiego 17, 80-214 Gdańsk, Poland; 3 Department of Urology, Medical University of Gdańsk, M. Smoluchowskiego 17, 80-214 Gdańsk, Poland; marcin.markuszewski@gumed.edu.pl (M.M.); marcin.matuszewski@gumed.edu.pl (M.M.)

**Keywords:** metabolomics, bladder cancer, urine, TURBT, time series analysis, metabolic fingerprinting, untargeted metabolomics

## Abstract

**Simple Summary:**

Bladder cancer is one of the most frequently diagnosed cancers worldwide and due to non-specific symptoms, it is often detected at a late stage. For this reason, possible diagnostic alternatives that could be used for non-invasive screening are still being sought. In recent years, metabolomics approach has been frequently used for this type of research, using urine or blood collected from two groups: patients with a given disease and healthy volunteers. Usually, to minimize the impact of between-subject differences, participants of the study are matched in terms of age, gender, or BMI. Another way to rule out the impact of this variability is to analyze samples taken at intervals from the same patient. Therefore, the aim of our study was to validate results obtained using the traditional approach on a small group of patients, from whom samples were taken before and after resection of the bladder tumor, in a given time frame.

**Abstract:**

The incidence of bladder cancer (BCa) has remained high for many years. Nevertheless, its pathomechanism has not yet been fully understood and is still being studied. Therefore, multiplatform untargeted urinary metabolomics analysis has been performed in order to study differences in the metabolic profiles of urine samples collected at three time points: before transurethral resection of bladder tumor (TURBT), the day after the procedure and two weeks after TURBT. Collected samples were analyzed with the use of high-performance liquid chromatography hyphenated with time-of-flight mass spectrometry detection (HPLC-TOF/MS) and gas chromatography coupled with triple quadrupole mass spectrometry detection (GC-QqQ/MS, in a scan mode). Levels of metabolites selected in our previous study were assessed in order to confirm their potential to differentiate the healthy and diseased samples, regardless of the risk factors and individual characteristics. Hippuric acid, pentanedioic acid and uridine confirmed their potential for sample differentiation. Based on the results of statistical analysis for the paired samples (comparison of metabolic profiles of samples collected before TURBT and two weeks after), a set of metabolites belonging to nucleotide metabolism and methylation processes was also selected. Longitudinal studies proved to be useful for the evaluation of metabolic changes in bladder cancer.

## 1. Introduction

Age-standardized incidence rates (ASR) of bladder cancer (BCa) increased from 5.3 per 100,000 in 2012 to 5.6 per 100,000 in 2020 [1,2]. This indicates that the incidence of BCa is not only not declining but is still a very pressing problem worldwide. Additionally, statistics show that the presence of at least one BCa risk factor increases the risk of developing the disease even several times. For example, about 90% of BCa cases are diagnosed in people over the age of 55. The risk of developing the disease is also more than four times higher in men than in women, and the proportion is similar in terms of mortality (3.2/100,000 in men and 0.9/100,000 in women). Smoking, the most important known BCa risk factor, increases the chance of developing the disease by about four times. In addition, it has been shown that cigarette smoking reduces the chances of recovery when cancer relapses and increases the risk of developing an invasive stage of the disease [2,3].

Each of the above-discussed risk factors, along with BMI value, accompanying diseases, etc., is also reflected in metabolic profiles determined by untargeted metabolomics analysis [4,5,6]. This is why it is so important to collect epidemiological data for any metabolomics-related experiment. In contrast to experiments with animals or microorganisms, research projects using human tissues or body fluids cannot be designed in a way that excludes the influence of non-studied factors on the metabolic fingerprint. There are three ways to design such a study involving human participants which are the most common. The first approach assumes minimizing the impact of age, gender, BMI and other important factors on the acquired profiles, by means of matching participants of studied groups on the basis of the collected epidemiological data. However, the selection of samples in this manner is very often impossible due to a restricted number of available study volunteers. There is also difficulty in collecting complete data on each participant (such as information about consumed drugs, dietary supplements, eating habits or stimulants). That is why the second approach, which requires inclusion of the complete set of epidemiological data in the model, is becoming more and more popular. All the collected data are introduced to the model to create the variables and explain the variability associated with them. Another way to minimize the influence of non-disease factors on the metabolic fingerprint (the third approach) is to schedule a longitudinal study [7,8,9,10]. This type of study allows the observation of participants over a period of time and aims to understand the mechanisms of a given change and of influencing factors.

In terms of metabolomics studies, BCa is quite an intensively studied disease entity [11,12]. However, a detailed pattern of development and progression of BCa is still not elucidated. This is why the main aim of the study was to assess changes in urinary metabolic profiles of samples obtained from BCa patients before and after resection of the bladder tumor, e.g., observation of disturbed energy metabolism, altered amino acid metabolism or elevated levels of modified nucleosides. High-performance liquid chromatography hyphenated with time-of-flight mass spectrometry detection (HPLC-TOF/MS) and gas chromatography coupled with triple quadrupole mass spectrometry detection (GC-QqQ/MS), in a scan mode) were utilized in order to obtain data sets covering metabolic profiles in a comprehensive manner. The studied group consisted of non-muscle-invasive BCa patients, from which the samples were taken at three time points: before transurethral resection of bladder tumor (TURBT), the day after TURBT was performed and at the follow-up visit two weeks after the resection.

The proposed study will enable a preliminary assessment of the differences in metabolite levels caused by the presence of a tumor. In addition, an evaluation of results obtained previously by our research group will be possible [13]. Briefly, comprehensive (by means of HPLC-TOF/MS, GC-QqQ/MS and proton nuclear magnetic resonance (^1^H NMR)) metabolomics analysis of urine samples obtained from healthy volunteers (*n* = 24) and patients diagnosed with muscle-invasive high grade BCa (*n* = 24) enabled the selection of seventeen metabolites-discriminating studied groups. A significant number of the selected compounds constitute the metabolites participating in energy and amino acid metabolism. The observation of previously selected metabolites will serve as a validation of previous results and help to determine which of them have the potential to distinguish healthy individuals from those with neoplastic changes, regardless the existing risk factors. Moreover, through the results of this project, it will be possible to select specific metabolites or biochemical pathways for targeted metabolomics analysis, carried out on a larger scale.

## 2. Materials and Methods

### 2.1. Characteristics of the Subjects Included into Study

All the subjects participating in the study were patients at the Urology Clinic of the Medical University of Gdańsk diagnosed with non-muscle-invasive bladder cancer (NMIBC). NMIBC accounts for approximately 70% of newly diagnosed cases [14]. Standard procedure in such a diagnosis involves transurethral resection of the bladder tumor (TURBT). TURBT constitutes a gold standard for NMIBC initial diagnosis, evaluation and treatment of choice [2,15]. TURBT is also used for resection of the tumor (aiming at complete resection if it is technically possible and safe for the patient). Risk stratification and treatment strategy is subsequently determined. In addition, due to high BCa recurrence rates and the risk that not all the neoplastic tissue is completely resected, TURBT is often repeated. All of the subjects were enrolled in a scheduled TURBT and were admitted to the hospital the day before the procedure.

The studied group consisted of 8 men and 2 women (due to the ASR proportions for women and men [1,2]). Participants were at the age of 69.6 (±5.6) and most of them smoked cigarettes (5 were current smokers, 4 had a long history of smoking and one had never smoked cigarettes). The average BMI value was 26.06 (±5.23). Four patients were of healthy weight, 3 were overweight and 3 were obese. All participants gave their informed consent to participate in the study. The presented study was also performed following the principles included in the Declaration of Helsinki and was approved by the Independent Committee of Bioethical Research at the Medical University of Gdańsk (numbers of consents: NKBBN/542/2017 and NKBBN/543/2017).

### 2.2. Samples’ Collection

**Figure 1:**
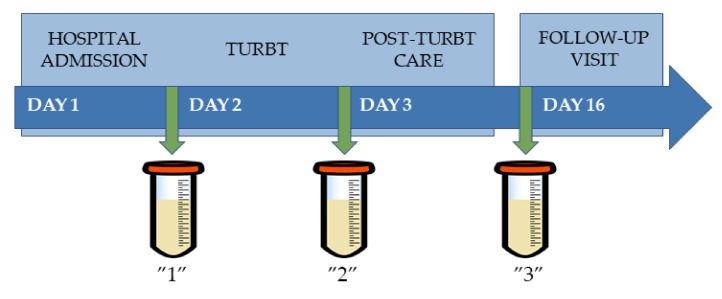
Scheme illustrating sample collection process. Legend: “1” (first time point, first morning urine samples collected on the day of TURBT, before the procedure), “2” (second time point, first morning urine samples collected the day after TURBT), “3” (third time point, first morning urine samples collected 14 days after TURBT).

Three samples were collected from each patient. First sample was collected on the morning of TURBT. Samples for the second time point were collected 1 day after the procedure. Third sample collection was scheduled for the follow-up visit, which was 2 weeks after TURBT. All samples were collected from the first morning urine, vortex-mixed for 15 s at 4 °C, transferred to tightly closed Eppendorf tubes and frozen at −80 °C. Simplified scheme illustrating samples’ collection process is presented in Figure 1.

An undoubted limitation of the presented research is the number of its participants. However, the premise of the project was that samples for all time points should be collected from each participant. Unfortunately, only about 20% of patients who were sampled at time points 1 and 2 (*n* = 53) showed up to the follow-up visit (within 2 weeks of discharge from the hospital, fasting, with the first morning urine collected). This situation was caused by the fact that for control visits patients often came to medical centers closer to their domicile, and could also be caused by the ongoing pandemic. This non-compliance also resulted in the inability to collect additional time points (after another 3 and 6 months).

### 2.3. Sample Preparation

#### 2.3.1. HPLC-TOF/MS

Thawed urine samples were vortex-mixed for 1 min and then centrifuged (2469× *g*, 15 min, 4 °C). Subsequently, 500 µL of deionized water (or acetonitrile in the case of analyses in HILIC mode) was added to 500 µL of urine sample. Then, samples were centrifuged (2469× *g*, 15 min, 4 °C) and filtered with the use of nylon filters (pore size 0.22 µm) directly to HPLC vials.

#### 2.3.2. GC-QqQ/MS

Thawed urine samples were vortex-mixed for 1 min and then centrifuged (2469× *g*, 15 min, 4 °C). Then, to 200 µL of supernatant, 50 µL of urease solution in water (600 units/1 mL) was added and samples were then incubated (37 °C, 30 min). Subsequently, deproteinization was performed by the addition of 800 µL of cold methanol (kept for at least 30 min in −80 °C) and 10 µL of solution of pentadecanoic acid in methanol (1 mg/mL) as internal standard was added. Samples were vortex-mixed (5 min) and centrifuged (2469× *g*, 15 min, 4 °C). Next, 200 µL of supernatants were transferred to glass inserts and evaporated to dryness (30 °C, 2 h). Two-step derivatization process was applied. First, 30 µL of methoxyamine in pyridine (15 mg/mL) was added. Samples were vortex-mixed (10 min) and incubated in a dark place (16 h, room temperature). As a second step, 30 µL of BSTFA with 1% TMCS was added and samples were vortex-mixed (5 min). Then, samples were incubated (1 h, 70 °C), 70 µL of heptane was added and samples were vortex-mixed (10 min) again.

#### 2.3.3. Quality Control Samples

Quality control samples (QCs) were obtained by pooling the same amount of all studied samples. The sample preparation procedures for QCs were identical to those for the real samples presented above.

### 2.4. Materials and Apparatus

#### 2.4.1. HPLC-TOF/MS

Analyses were performed with 1200 HPLC 6224 TOF/MS system (Agilent Technologies, Waldbronn, Germany) with dual electrospray ionization source (Dual-ESI). Compound separation was possible due to Zorbax Extend-C18, Rapid Resolution HT column (2.1 × 100 mm; 1.8 µm) and Poroshell 120 HILIC column (4.6 mm × 50 mm; 2.7 μm) application. The injection volume was set to 2 µL. In case of analysis in RP mode, mobile phase flow rate and time of analysis were set to 0.35 mL/min and 18 min, respectively. Mobile phase (A—0.1% formic acid in water and B—0.1% formic acid in acetonitrile) gradient program was: 3–6 min from 98% to 80% of A, 6–9 min from 80% to 55% of A, 9–14 min from 55% to 2% of A and from 14–18 min 2% of A. The column was equilibrated for 10 min and the column temperature was set to 35 °C. In case of analysis in HILIC mode, mobile phase flow rate and time of analysis were set to 0.4 mL/min and 15 min, respectively. Mobile phase (A—10 mM ammonium buffer (pH 3.44) and B—acetonitrile) gradient program was: 3–6 min from 30% to 35% of A, 6–9 min from 35% to 40% of A, 9–12 min from 40% to 45% of A and from 12–15 min 45% of A. The column was equilibrated for 7 min, and its temperature was set to 25 °C. The mass spectrometer for LC-MS analyses was operated in a scan mode with the *m*/*z* range from 50 to 1100, in both positive and negative ionization modes. The capillary and fragmentor voltage were 3250 V and 150 V, respectively.

#### 2.4.2. GC-QqQ/MS

Analyses were performed with the GC TQ8030 system (Shimadzu, Kyoto, Japan) equipped with electron ionization (EI) ion source and Zebron ZB-5MS column (30 m × 0.25 mm, 0.25 μm; Phenomenex, Torrance, CA, USA) with helium as a carrier gas. Sample injection volume was 1 µL and the injector temperature was set to 250 °C. The temperature gradient program was: 60 °C (1 min), 60 320 °C (8 °C/min), 320 °C (5 min), with a total analysis time of 38.5 min. The mass spectrometer was operated in a scan mode with the *m*/*z* range from 50 to 600. The ion source voltage and temperature were set to 70 eV and 200 °C, respectively. At the beginning of the sequence run, the mixture of alkanes (from C10 to C40, even carbon number) was analyzed, as it is required for retention indices calculation, retention time alignment and metabolite identification.

### 2.5. Data Processing and Statistical Analysis

#### 2.5.1. Data Pretreatment

The LC-MS data pretreatment procedure included deconvolution of all collected analytical signals with the use of the Molecular Feature Extraction (MFE) algorithm in MassHunter Qualitative Analysis software version B.04.00 and DA Reprocessor B.04.00 (B371) from Agilent Technologies. As a next step, feature extraction was performed based on charge value, isotopic pattern and presence of dimers and adducts (+H, +Na in positive ionization mode; -H, +HCOO in negative ionization mode; and neutral water loss -H_2_O). Subsequently, Mass Profiler Professional B. 02.01 software (MPP; Agilent Technologies, Santa Clara, CA, USA) was used for feature alignment, with 1% shift in the retention time and 20 ppm error in measured mass considered as acceptable. The data filtration step was based on the quality assurance criteria (presence in 50% of QC samples, coefficient of variance (CV) in QCs lower than 20%) [16,17].

For GC-MS data deconvolution and identification the Automated Mass Spectral Deconvolution and Identification System (AMDIS; National Institute of Standards and Technology, Gaithersburg, MD, USA) was used. Retention indices (RI) calculation, retention time (RT) alignment (applied RT window: ± 0.1 min) and compound identification based on the NIST11 spectra library were performed. Similarly to the LC-MS, data filtration was performed using Mass Profiler Professional B.02.01 (MPP; Agilent Technologies, Santa Clara, CA, USA). Quality assurance criteria included presence of the metabolite signal in 50% of QC samples, CV in QCs < 30%.

**Figure 2:**
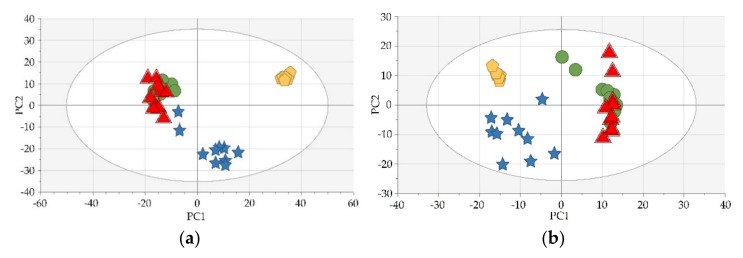
Exemplary score plots of the Principal Component Analysis models (built with the use of SIMCA-P+ 13.0.3 (Umetrics, Umeå, Sweden)) presenting the clustering of QCs. The clustering assessment was made in order to verify systems stability for analysis using (**a**) LC-HILIC-MS in positive ionization mode and (**b**) LC-RP-MS in positive ionization mode. Legend: green circles—“1” (first time point, samples collected before TURBT), blue stars—“2” (second time point, samples collected the day after TURBT), red triangles—“3” (third time point, samples collected 14 days after TURBT), yellow pentagons—QCs. The number of samples: “1” = 10, “2” = 10, “3” = 10, QCs = 7.

After assessment of systems stability (Figure 2), additional data filtration was performed. For LC-MS data, features for which a minimum of 80% pairs of results were obtained between the 1st and 3rd time points were selected and for GC-MS: 70%. Afterwards, signal intensities were normalized with the use of specific gravity (SG) value. SG, along with creatinine (CR) concentration, is commonly used to correct measured levels of metabolites in urine samples. Their unquestionable advantage is the low cost of the test or high data availability. However, due to the fact that CR concentration may vary due to age, race or gender of the patient, SG was our normalization method of choice [18,19].

#### 2.5.2. Metabolites Annotation

Annotation of metabolites detected with the use of the LC-MS technique was performed with the use of a freely available tool for their overview—CEU Mass Mediator version 3.0 (http://ceumass.eps.uspceu.es/ accessed on 6 December 2021) [20]. It corresponds to level 2 of confidence system for identification of metabolites for studies concerning untargeted metabolomics by Metabolomics Standards Initiative. In classification proposed by Schrimpe-Rutledge et al. it would fit in level 3 [21].

In case of GC-MS technique, compounds’ tentative identification was performed by comparison of the obtained MS spectra with NIST11 spectra library, with the use of AMDIS software (level 2).

#### 2.5.3. Semi-Targeted Analysis

For semi-targeted analysis, LC-MS data were processed using MassHunter Profinder B.06.00 software (Agilent Technologies, Santa Clara, CA, USA). The Batch Targeted Analysis option was used with the compounds library, built based on previously published results [13]. The library included both compound formula and molecular mass. During data inspection the quality of the MS spectra was verified as well as the comparison of metabolites retention times with those obtained previously was made. Visual inspection of the mass spectra allowed the elimination of false-positive results in the created data matrix.

For GC-MS data the compounds were selected from data matrix prepared for untargeted analysis.

Selected signal intensities (from LC-MS and GC-MS analyses) were normalized in three ways: with the use of SG values, creatinine (CR) concentration (both measured with the use of portable urine analyzer: Urit 31, URIT Medical Electronic Co., Guangxi, P.R. China) or by using the abundance of creatinine MS signal obtained during analyses. A comparison of results of statistical analysis performed on those 3 datasets can show whether results will differ depending on the selected method of normalization.

## 3. Results

### 3.1. Semi-Targeted Metabolomics Analysis

**Figure 3:**
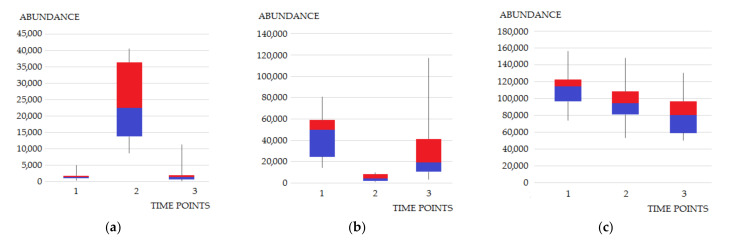
Differences in metabolite abundance at three time points for (**a**) propanoic acid, (**b**) meso-erythritol, (**c**) uridine. Signal intensities were normalized by SG. *p*-values, indicating the statistical significance of the differences in abundance between time points “1” and “3”, for propanoic acid, meso-erythritol and uridine were 0.52, 0.22 and 0.02, respectively. Legend: Line segments, blue and red boxes represent consecutive quartiles. Time points: “1”—samples collected before TURBT; “2”—samples collected the day after TURBT; “3”—samples collected at follow-up visit (14 days after TURBT). The number of samples: “1” = 10, “2” = 10, “3” = 10.

In the first stage, levels of previously chosen metabolites (which have shown statistically significant differences between BCa patients and healthy individuals in the previous study [13]) were assessed. Some of the differences in the intensity of the signals at three time points were immediately apparent (Figure 3). It was observed that the levels of some metabolites determined the day after TURBT (second time point) clearly differed both from the state before surgery and two weeks after TURBT. Urine levels of propanoic acid had increased more than tenfold the day after surgery, but had decreased back after two weeks (Figure 3a). Similar observations were made for 2-deoxy-ribonic acid and benzenediol. On the other hand, the level of meso-erythritol determined at the second time point decreased several times in comparison to the first one. At the third time point, its level approached the state from before TURBT. Moreover, the level of S-adenosylmethionine were too low to be determined in the first and third time points, and a signal derived from this compound was only observed in samples collected at the second time point. The observed differences might be directly associated with the performed procedure and administered medications, changed diet related to hospitalization or inflammation caused by the procedure. Such observations also assured us that further comparisons should be made between the first and third time points.

Processed semi-targeted data were subjected to three normalization strategies (Table 1). In order to evaluate the differences in metabolites abundance, univariate test for paired measurements was applied. Based on its results (Table 1), 3 metabolites were selected as those that show the greatest potential for differentiating neoplastic samples from healthy ones, namely: hippuric acid, pentanedioic acid and uridine (*p*-value ≤ 0.05). Additional attention could be paid also to diacetylspermine, glutamine, phenylacetylglutamine and uric acid (*p*-value ≤ 0.10, for at least 2 normalization strategies).

Metabolites, differentiating the studied time points the most, belong to amino acid metabolism. Due to the fact that amino acids play various roles in the tumor and its microenvironment, their reprogrammed metabolism in cancer development and progression has been reported many times [22,23]. The role of amino acids in cell proliferation was also highlighted in the case of BCa [24]. In brief, the elevated level of pentanedioic acid (glutaric acid) might be due to a disturbed metabolism of lysine or tryptophan. Pentanedioic acid is known for its action as an acidogen and metabotoxin, when present in high concentrations [25]. Differences in hippuric acid level might be associated with disturbances in phenylalanine metabolism, associated with the increased energy demand of proliferating cells, and were previously observed in urine of BCa patients [26]. However, one must also take into account the fact that altered levels of hippuric acid might occur due to microbiome- or diet-related changes. Altered levels of uridine supports the thesis of enhanced metabolism of nucleic acids, hence the overactivity of cancer cells. A more detailed description of the changes in the levels of all the above-mentioned metabolites and their possible explanation was presented in our previous study [13]. The observed changes confirm previous reports and induce further examination of the concentrations of the selected metabolites in urine samples.

### 3.2. Untargeted Metabolomics Analysis

Features selected during data pretreatment, with a sufficiently high percentage of complete pairs of measurements (for the first and third time points), were subjected to univariate statistical analysis for paired measurements. Over one hundred features met the criteria of statistical significance of differences between the first and third time points. More than 20 of them were annotated (see Section 2.5.2), narrowing the variables to those in which the error related to the mass measurement was less than 5 ppm. Such data selection allowed the analysis of metabolic pathways and biochemical considerations. Detailed characteristics of differences in levels of annotated metabolites is presented in Table 2.

As seen in Table 2, a great number of metabolites, differentiating urine samples collected before TURBT and those collected two weeks after the procedure, belong to amino acid metabolism. Moreover, the differences observed in the conducted study confirm previously observed changes in the metabolic pathways related to purine and pyrimidine metabolism. Most of the metabolites whose levels were observed as altered in the study are molecules that take part in processes required for extensive growth and proliferation of BCa cells [27].

## 4. Discussion

Purines and pyrimidines participate in a wide range of biological processes, e.g., in cell proliferation [28]. Their abnormal metabolism may point to tumor development or progression [29,30]. Elevated levels of methylguanine, observed in the presented study, might be associated with enhanced nucleic acid turnover or their intensified methylation. An increased presence of methyladenosine, one of the most common modifications of RNA, was also previously reported in cancer [31,32], including BCa [33]. Its important role in tumorigenesis and the progression of cancer was described in detail by He et al. [32].

**Figure 4:**
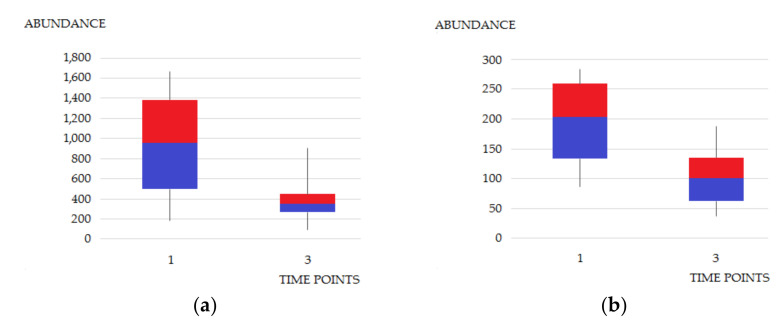
Differences in metabolite abundances at time points “1” (samples collected before TURBT) and “3” (samples collected 14 days after TURBT) for (**a**) 1,3-dimethyluracil and (**b**) N1-methyl-2-pyridone-5-carboxamide. Signal intensities were normalized by SG. *p*-values, indicating statistical significance of the differences in abundance between time points “1” and “3”, for 1,3-dimethyluracil and N1-methyl-2-pyridone-5-carboxamide were 0.02 and 0.01, respectively. Legend: Line segments, blue and red boxes represent consecutive quartiles. Time points: “1”—samples collected before TURBT; “2”—samples collected the day after TURBT; “3”—samples collected at follow-up visit (14 days after TURBT). The number of samples: “1” = 10, “2” = 10, “3” = 10.

It can be noticed that methylated metabolites account for almost half of the annotated compounds. Altered levels of many of them have already been reported in various types of cancer and other diseases [34,35,36,37,38,39]. The obtained results are also in line with previous reports of abnormal levels of urinary nucleosides during pathophysiological conditions, e.g., urinary tract cancers and breast cancer [34]. It can be seen that levels of dimethylated uracil (Figure 4a) are significantly elevated in the first time point compared to the third one. The same applies to N1-methyl-2-pyridone-5-carboxamide (Figure 4b), which is a metabolite of nicotinamide and its intermediate metabolite, methylnicotinamide (MNA). The mechanism of action of nicotinamide N-methyltransferase and the overproduction of MNA are important aspects of cancer cell function and have been intensively studied by Ulanovskaya et al. [39].

**Figure 5:**
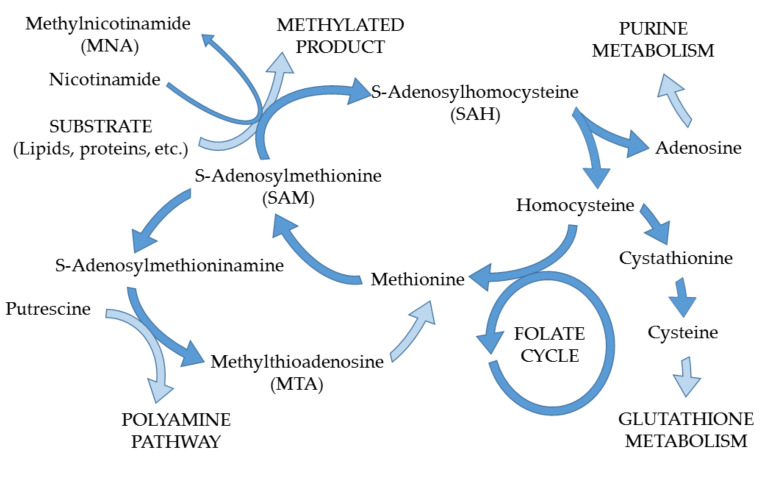
Scheme illustrating methylation process, potentially enhanced during BCa, along with related metabolic pathways. Based on [35,36,39].

As the methylation process indicates the presence and intensity of metabolic aberrations, the potential for the determination of methylated metabolites in metabolomics studies was recognized and resulted in the development of a method for their quantitation [40]. Furthermore, it was recently applied for BCa cells and confirmed their up-regulation in cancer. The potential role of methylated metabolites as diagnostic markers of BCa was highlighted in several studies (as a result of intensified processes of DNA methylation) [41]. The mechanism of alterations in methyl metabolism and related pathways is shown schematically in Figure 5.

It is worth noting the fact that few of the determined metabolites (N-acetylneuraminic acid, creatine riboside or indoxyl sulfate) were previously reported as possible markers of intrahepatic cholangiocarcinoma, lung cancer or adrenal neoplasms [42,43,44]. In addition, the elevated level of glutarylcarnitine in cancer is consistent with the observations regarding the increased level of pentanedioic acid [13].

## 5. Conclusions

The presented preliminary study showed that the implementation of longitudinal data in metabolomics analysis is of great importance and provides valuable information. The conducted study enabled the validation of results of previous experiments and the selection of metabolites with the highest potential for differences between urine levels in healthy and BCa patients, regardless of the risk factors and inter-individual differences. Taking into account the observed differences in the levels of putatively annotated metabolites, as well as our previous findings, it will be most meaningful to focus the next step on amino acids, nucleotides metabolism and methylation process. However, being aware of the limitations of our study, collecting a larger pool of samples and additional time points for each subject would allow for a more accurate assessment of the observed changes and would be preferred for further research.

## Figures and Tables

**Table 1:** A summary of the significance of differences in metabolite levels (point 1 vs. point 3) and resulting from applied normalization strategy. Signal intensities were normalized by specific gravity or creatinine level; SG—specific gravity; CR (PUA)—creatinine level measured with the use of portable urine analyzer; CR (MS)—creatinine signal measured with the use of LC-TOF/MS.

Metabolite	*p*-Value		
	SG	CR (PUA)	CR (MS)
Benzenediol	0.78	0.76	0.75
2-deoxy-ribonic acid	0.67	0.83	0.59
Diacetylspermine	0.08	0.10	0.08
Meso-erythritol	0.22	0.17	0.19
Glutamine	0.06	0.06	0.07
Hippuric acid	0.03	0.07	0.07
Lactic acid	0.72	0.78	0.46
Pentanedioic acid	0.06	0.11	0.02
Phenylacetylglutamine	0.06	0.08	0.11
Pipecolic acid	0.07	0.15	0.11
Propanoic acid	0.57	0.99	0.55
Threonic acid	0.10	0.12	0.12
Tyrosine	0.57	0.68	0.59
Uric acid	0.09	0.07	0.06
Uridine	0.02	0.04	0.07

**Table 2:** A summary of the significance of differences in annotated metabolite levels (before TURBT (point 1) vs. after TURBT (point 3)). Signal intensities were normalized by SG and scaled.

Metabolite	*p*-Value	Average Signal Intensity		Diff	SD Diff
		Before TURBT	After TURBT		
N-Acetylneuraminic acid	0.02	0.58	0.31	0.27	0.30
Androsterone 3-glucuronide	0.03	0.35	0.25	0.10	0.11
Creatine riboside	0.01	0.48	0.19	0.29	0.25
Creatinine	0.05	0.51	0.27	0.24	0.27
5,6-Dihydrouridine	0.03	0.55	0.30	0.25	0.26
N6,N6-Dimethyl-L-Lysine	0.04	0.43	0.28	0.15	0.20
1,3-Dimethyluracil	0.02	0.54	0.19	0.35	0.39
Glucosylgalactosyl hydroxylysine	0.04	0.33	0.18	0.15	0.17
Glutarylcarnitine	0.02	0.32	0.11	0.21	0.24
Guanidinosuccinic acid	0.03	0.47	0.22	0.25	0.26
Indolelactic acid	0.02	0.24	0.16	0.08	0.10
Indoxyl sulfate	0.01	0.32	0.18	0.14	0.14
N6-Methyladenosine	0.01	0.47	0.23	0.24	0.24
3-Methylglutarylcarnitine	0.02	0.34	0.10	0.24	0.27
1-Methylguanine	0.04	0.52	0.31	0.21	0.27
1-Methylinosine	0.02	0.38	0.19	0.19	0.19
N6-Methyl-L-Lysine	0.04	0.62	0.33	0.29	0.38
Succinylcarnitine ^1^	0.01	0.51	0.27	0.24	0.22
N-Methylnicotinamide	0.04	0.44	0.23	0.21	0.28
N1-Methyl-2-pyridone-5-carboxamide ^2^	0.01	0.64	0.22	0.42	0.32
L-Glutamic acid	0.04	0.64	0.40	0.24	0.32
O-Sebacoylcarnitine	0.03	0.54	0.17	0.37	0.34
Succinyladenosine	0.02	0.50	0.26	0.24	0.22
Tryptophan	0.02	0.25	0.16	0.09	0.10
L-Valine ^3^	0.04	0.50	0.18	0.32	0.37

^1^ /Methylmalonylcarnitine; ^2^ /N1-Methyl-4-pyridone-3-carboxamide; ^3^ /Norvaline.

## Data Availability

The data presented in this study are available on request from the corresponding author. The data are not publicly available due to confidentiality of the data.
